# Familial Subcutaneous Granuloma Annulare: A Case Report of Two Siblings

**DOI:** 10.7759/cureus.71298

**Published:** 2024-10-12

**Authors:** Anouschka Kul, Axel De Greef, Marie Baeck, Evelyne Harkemanne

**Affiliations:** 1 Department of Dermatology, Cliniques universitaires Saint-Luc (UCLouvain), Brussels, BEL

**Keywords:** familial form, generalized, granuloma annulare, inflammatory granulomatous disorder, subcutaneous

## Abstract

Granuloma annulare (GA) is a rare inflammatory granulomatous disorder and its subcutaneous forms are even more uncommon. Only a few familial cases of this condition have been reported in the literature. We report a case of familial subcutaneous GA in two siblings during childhood.

## Introduction

Granuloma annulare (GA) is a benign inflammatory granulomatous skin disease, and its most common clinical presentation involves erythematous annular papules and plaques [1]. The most frequent locations are the extremities, mainly the hands and feet [2]. This disease is classified into two clinical subtypes: localized and generalized forms [1]. Atypical forms include perforating and subcutaneous GA [2,3]. Perforating GA presents as umbilicated papules with central crusting and scarring, while the subcutaneous form manifests as firm nodules [4]. While its etiology is unknown, T-helper cytokines are presumed to play an important role [2]. Rare cases of familial forms have also been described [4]. In this report, we present the case of two sisters with subcutaneous GA.

## Case presentation

**Figure 1:**
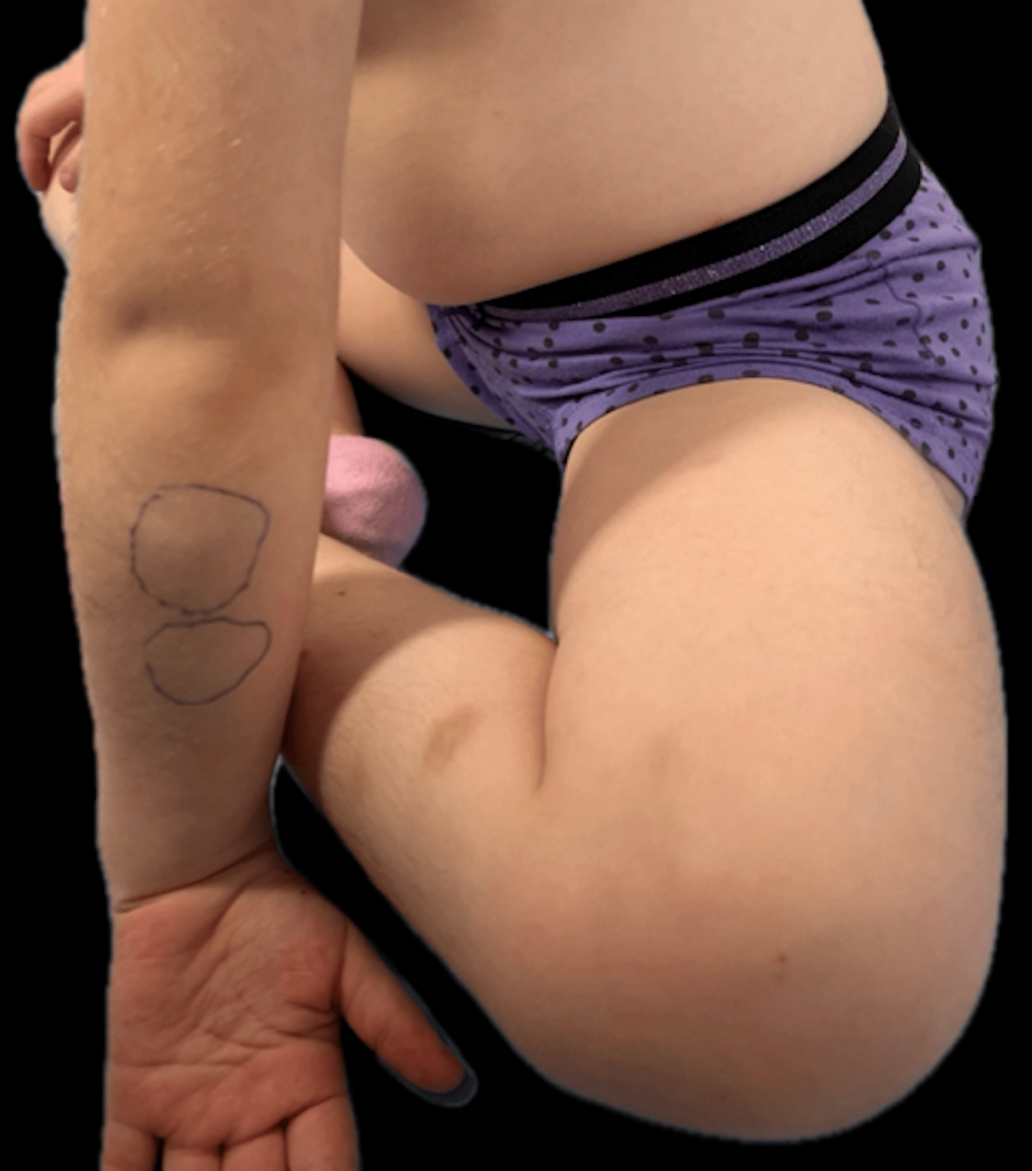
Clinical photograph of two subcutaneous, adherent, non-mobile nodules on the left forearm of the five-year-old sister

A five-year-old female presented with asymptomatic rapidly growing subcutaneous nodular lesions of the limbs that had first appeared three months ago. She was otherwise healthy with no particular medical history. On clinical examination, three subcutaneous, adherent, non-mobile nodules were noted on the left forearm and the right lower limb (Figure 1).

**Figure 2:**
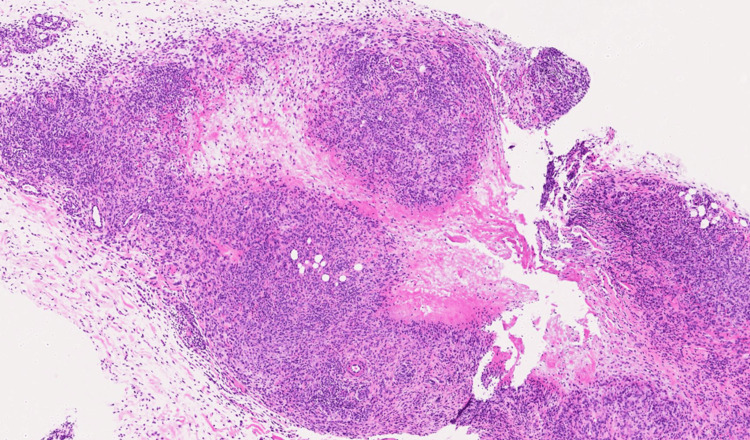
The histopathological image showed eosinophilic, loose, and myxoid material, punctuated by nuclear debris and surrounded by palisading histiocytes Hematoxylin-eosin, magnification 30x

The lesions were poorly defined and non-pulsatile. Ultrasound exploration showed irregular hypervascularized hypoechogenic areas within the hypodermis. Given the relatively rapid growth and hypervascularization, several differential diagnoses were suggested, such as cutaneous lymphoma, sarcoidosis, epithelioid sarcoma, and GA. A biopsy was therefore performed. Histology showed an eosinophilic, loose, and myxoid material, punctuated by nuclear debris and surrounded by palisading histiocytes (Figure 2). 

**Figure 3:**
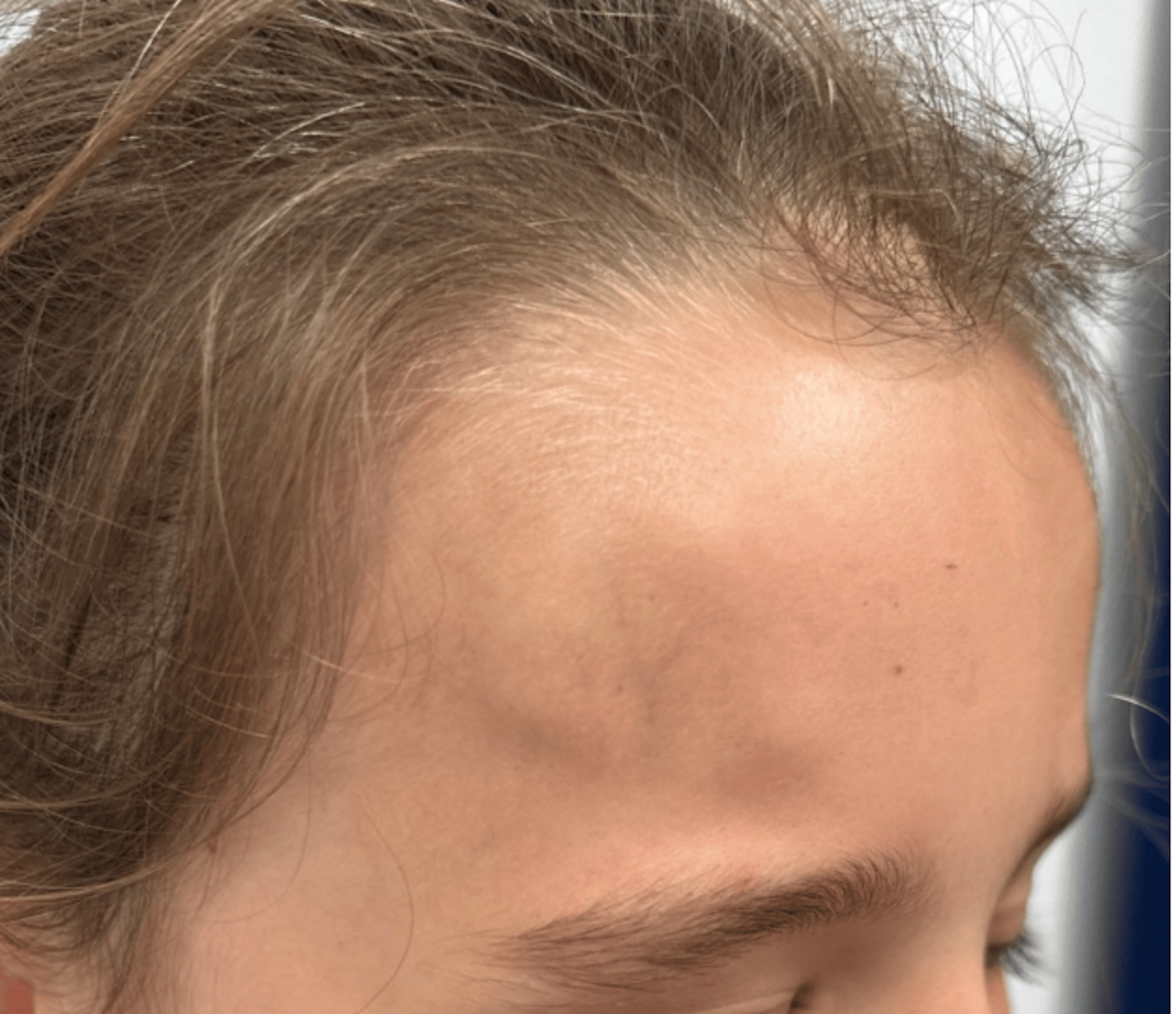
Clinical photograph of the three subcutaneous ring-shaped nodules on the forehead of the nine-year-old sibling

Periodic Acid-Schiff (PAS) and Wade-Fite staining were negative. Immunohistochemistry was also performed: cytokeratins AE1/AE3 were negative and BAF47 showed no loss of nuclear expression. These histological features were consistent with subcutaneous GA. Interestingly, the lesions disappeared spontaneously a few weeks after the biopsy. Four months later, the patient's nine-year-old sister presented with three subcutaneous ring-shaped nodules on the forehead (Figure 3). 

Ultrasound examination showed subgaleal irregular hypoechogenic areas. As the clinical appearance was highly suggestive of GA, we decided not to perform a skin biopsy.

## Discussion

The global incidence of GA is around 0.04% [2]. Familial GA, despite being rarer, has been reported in siblings or successive generations in the form of different subtypes: localized nodular, generalized perforating, and generalized or localized ring-shaped papules [5-8]. Our cases were very unusual given the occurrence of the subcutaneous form of familial GA in two siblings almost simultaneously during childhood. The generalized subcutaneous subtype of this condition is rare. It is characterized by painless indurated nodules and is more frequently found in children [4,7]. Lesions are more frequent on the limbs, scalp, and hands [7]. Histology usually shows palisading granulomatous inflammation of the deep dermis and subcutaneous tissue [4].

The etiopathology of the disease is not yet fully understood [5,6], although recent studies have reported the involvement of the T-helper 1, 2, 17, and 22 cytokines and the activation of Janus kinase signal transducer and activator of transcription pathway [2]. In addition, different HLA associations with a higher prevalence of HLA-A29 in localized forms of GA and HLA-B35 in generalized forms have been reported [5,6]. These associations could indicate a genetic predisposition and need to be further investigated [6]. Potential triggers of these cell-mediated reactions have been proposed, such as infections, which could possibly explain familial GA with a close timeline [2,5,6].

Treatment is not standardized, but topical and intralesional injections of corticosteroids are the first-line therapy for localized subtypes [9,10]. For generalized forms, systematic treatments such as methotrexate, antimalarials, isotretinoin, dapsone, as well as Janus kinase inhibitors have been proposed [2,6,9,10]. The prognosis is excellent, and the response to treatment depends on the subtype, with localized forms generally responding better than generalized forms [10]. However, spontaneous resolution of the lesions is common and sometimes enhanced by surgical procedures [11], as in the case of the first sister in this report.

## Conclusions

Subcutaneous GA is uncommon. However, its diagnosis should be considered in the presence of single or multiple, skin-colored, ring-shaped, asymptomatic subcutaneous nodules appearing most often on the limbs. Although rare, familial GA does exist and can occur in both adults and children. Our case report is unique in that it describes the subcutaneous form of familial GA occurring in two siblings almost simultaneously during childhood. The exact pathophysiological mechanisms of these forms are still unknown. Given the indolent nature of the disease and the possibility of spontaneous resolution, treatment can be initially withheld and a conservative approach can be suggested.
